# Mild Cognitive Impairment Diagnosis and Care Algorithms and the Role of Digital Interventions: Expert Consensus

**DOI:** 10.1002/cns.71085

**Published:** 2026-09-02

**Authors:** Nagaendran Kandiah, Yee Fai Chan, Darwin Dasig, Jacqueline Dominguez, Kar Chai Koh, Si Ching Lim, Paulus Anam Ong, Encarnita Raya‐Ampil, Raymond Chee Seong Seet, Vorapun Senanarong, Kit Mun Tan, Maw Pin Tan, Trần Công Thắng, Tong Mai Trang, Yuda Turana, Cuibai Wei, Ralf Ihl

**Affiliations:** ^1^ Dementia Research Centre Singapore Singapore; ^2^ Nanyang Technological University Singapore Singapore; ^3^ Hospital Kuala Lumpur Wilayah Persekutuan Kuala Lumpur Malaysia; ^4^ Makati Medical Centre Makati Philippines; ^5^ St Luke's Medical Center Bonafacio Global City Makati Philippines; ^6^ Poliklinik Kepong Baru Wilayah Persekutuan Kuala Lumpur Malaysia; ^7^ Changi General Hospital Singapore Singapore; ^8^ Hasan Sadikin Hospital Bandung Indonesia; ^9^ University of Santo Tomas Manila Philippines; ^10^ Yong Loo Lin School of Medicine National University of Singapore, and National University Hospital Singapore Singapore; ^11^ Faculty of Medicine Siriraj Hospital Mahidol University Bangkok Thailand; ^12^ Universiti Malaya Wilayah Persekutuan Kuala Lumpur Malaysia; ^13^ University Medical Center HCMC Ho Chi Minh City Vietnam; ^14^ School of Medicine and Health Sciences Atma Jaya Catholic University of Indonesia North Jakarta Indonesia; ^15^ Xuanwu Hospital Capital Medical University Beijing China; ^16^ University of Duesseldorf Düsseldorf Germany

**Keywords:** dementia, geriatrics, *Ginkgo biloba*, memory disorders, mild cognitive impairment, neurocognitive disorders

## Abstract

**Background:**

Mild cognitive impairment (MCI) is a clinically recognized condition, often being a prodromal dementia state with a risk of further cognitive decline. While there is currently no definitive diagnostic test for MCI, early identification and intervention are important.

**Methods:**

To assist clinicians in the diagnosis and treatment of MCI, the ASian Clinical Expert group on Neurocognitive Disorders (ASCEND) convened to identify ongoing gaps in MCI management primarily in Asia, and to discuss the existing body of literature with a view to providing evidence‐based expert recommendations.

**Results:**

Two novel algorithms and a set of expert recommendations were formulated, addressing the cognitive assessment and clinical diagnosis of MCI, and optimal MCI management approaches. Because many risk factors for cognitive decline are modifiable, the mainstay of intervention comprises a multi‐domain approach involving optimizing metabolic health, mitigating cerebrovascular risk, correcting hearing and vision loss, treating depression, encouraging engagement in cognitive exercises and social connection, and avoiding harmful substances. Digital interventions are becoming increasingly important as tools for cognitive assessment, as well as for cognitive stimulation and to help patients self‐manage and maintain their independence. Pharmacologic approaches for MCI management include medical management of cardiovascular risk, reducing cholinergic burden and the use of drugs known to sedate or affect cognitive function, and evidence‐based medications that have demonstrated benefit specifically in MCI.

**Conclusions:**

These evidence‐based recommendations are intended to support clinicians, particularly in primary care, to identify MCI and plan an intervention strategy to help slow age‐related cognitive decline.

## Graphical abstract

Mild cognitive impairment (MCI) carries a risk of further cognitive decline; thus, early diagnosis and intervention are important. The ASCEND Expert Group presents novel algorithms for (1) cognitive assessment/clinical diagnosis and (2) MCI management, emphasizing a multimodal strategy encompassing metabolic/cerebrovascular risk reduction, sensory loss correction, psychosocial interventions, cognitive stimulation (including digital interventions), and evidence‐based pharmacotherapy.

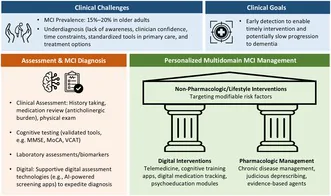

## 1 Introduction

In this rapidly evolving digital era, technology is fundamentally transforming healthcare delivery and patient management. Digital tools are becoming indispensable assets for both clinicians and patients, enhancing clinicians' ability to detect, monitor, and manage a range of conditions with greater accuracy and efficiency and empowering patients to take greater control of their own care and maintain their independence. This technological revolution may be particularly relevant in the realm of geriatric care, where age‐related cognitive decline presents complex diagnostic and management challenges. As the population of older adults becomes increasingly conversant with smartphones, tablets, and digital interfaces, innovative digital tools represent an important opportunity to facilitate earlier detection of cognitive impairment as well as potentially offering a cost‐effective and personalized approach to monitoring and management.

Mild cognitive impairment (MCI) is a clinically recognized intermediate neurocognitive state between normal cognitive aging and dementia, with evidence of cognitive decline but without appreciable impact on an individual's activities of daily living (ADL) [1]. In addition to cognitive and memory symptoms, patients with MCI often display neuropsychiatric symptoms (NPS), including irritability, depression, anxiety, apathy, or behavioral changes, which are observed in around half of cases and can exacerbate the burden of disease [1, 2]. As with dementia, MCI (which can be a prodromal stage of dementia) may have multiple causal subtypes due to multifactorial pathophysiology [3, 4]. For example, Alzheimer's disease (AD), the most common form of dementia, can arise secondary to other disease processes, including neurologic, neurodegenerative, cerebrovascular, or psychiatric disorders [5].

There are few official estimates of MCI prevalence in older adults in Asian populations, because of varying definitions, diagnostic criteria, populations, cultural aspects (including unwillingness to seek medical care for memory and cognitive issues), and a general lack of awareness of early symptoms [6]. Globally and in Asia, estimates of MCI prevalence are around 15%–20% [1, 5]. MCI prevalence increases with age; a global meta‐analysis estimated that around 7% of individuals aged 60–64 are living with MCI, increasing to 15% in those aged 75–79 and to 25% in those aged 80–84 years [5].

In addition to age, other factors that may increase the risk of cognitive decline include low education, physical inactivity, midlife hypertension, midlife obesity, diabetes, high LDL cholesterol, smoking, depression, alcohol consumption, hearing and vision loss, traumatic brain injury, social isolation, and air pollution [7, 8, 9]. Consistent with the presence of cardiovascular risk factors, cerebrovascular disease is thought to be underdiagnosed and underestimated as a potential contributor to cognitive decline [10].

Individuals with MCI are at increased risk of further progression to dementia. The annual conversion rate is estimated at approximately 10%–20% per year, compared to around 1%–2% dementia incidence in the general older population [11, 12]. Some individuals with MCI (around 15%–40% annually) revert to normal cognitive function, but this group remains at higher risk of MCI re‐diagnosis and ultimately dementia [5, 13]. Risk factors reported to increase the likelihood of progression include advancing age, vascular disease and risk factors, markers of cerebrovascular disease, diabetes mellitus, baseline memory impairment, NPS, depressive symptoms, and impaired executive performance [14].

Because many of the risk factors for cognitive decline are potentially modifiable, multiple opportunities exist for intervention. Early identification of MCI—and particularly of patients at higher risk of progression—can enable timely intervention and optimize the chance of slowing cognitive decline and dementia onset [15]. US population modeling suggests that a modest 2‐year delay in dementia onset could reduce the number of persons living with dementia by almost 20% over 20 years [16]. This is of critical importance given the massively escalating burden of cognitive disorders on national healthcare systems [6, 17], as well as the individual quality of life (QoL) and psychosocial burdens of cognitive decline on patients and their caregivers [1, 5].

Published and anecdotal evidence across primary care and physician specialties indicates broad variation in MCI assessment and management approaches, both within Asia and beyond, and suggests that dedicated initiatives are needed to improve general understanding of MCI and standardize its diagnosis and management [6, 18]. To this end, herein the ASian Clinical Expert group on Neurocognitive Disorders (ASCEND) reviews the relevant evidence from the body of literature and presents newly consolidated diagnosis and management algorithms, offering expert consensus recommendations to guide clinicians through MCI diagnosis and management.

## 2 Methods

The ASCEND Expert Group comprises around 20 invited members primarily from the Asia‐Pacific (APAC) region. First established in 2017, ASCEND includes selected interdisciplinary experts from neurology, geriatrics, psychiatry, and pharmacy and aims to improve the awareness, diagnosis, and management of MCI and dementia through research, evidence‐sharing, and expert recommendations. ASCEND has previously published two consensus statements on the treatment and management of MCI and dementia [4, 19].

Building on the success of prior initiatives, 17 members of the ASCEND Expert Group from 8 countries (Singapore, Malaysia, Philippines, Indonesia, Thailand, Vietnam, China, and Germany) reconvened in August 2024. The key purpose was to further discuss ongoing gaps in MCI diagnosis and management in Asia, and work toward practical solutions to meet these needs. A predefined objective of the meeting was to develop a plan to address existing gaps across the region by promoting early diagnosis and intervention—in particular, through exploring the emerging role of digital diagnostics and multidomain digital interventions as part of an optimal MCI management approach.

A 7‐question pre‐meeting survey was conducted among the group to gather information about (1) current challenges in diagnosing and managing MCI in Asia, (2) the potential utility of digital tools, and (3) the experts' recommendations for practice improvements including a digital component. In parallel, a relevant literature review was conducted using standard, non‐systematic search methodology on a large publicly available database (PubMed) and an AI research tool (Consensus AI). These searches provided a broad and thorough overview of global MCI diagnosis and treatment approaches, including the utility of digital interventions. During the meeting, the ASCEND Expert Group collectively discussed the pre‐meeting survey results, listened to presentations from key opinion leaders from Asia and Germany, and critically assessed the literature findings. Based on the aggregated recorded and written outcomes from this meeting, a series of consensus statements and two algorithms were developed by an independent editorial assistance team. These consensus statements were then critically reviewed by each ASCEND expert independently. Through three rounds of shared (name‐attributed) email discussion from May through October 2025, the group qualitatively discussed each proposed statement, with the opportunity to explain their responses. Between rounds, responses and reasoning were collated and adjudicated by the editorial assistance team and were circulated to the group. No author raised any substantive objections at any stage, and the group ultimately arrived at unanimous consensus after only minor wording changes to several statements. This iterative consensus process was successfully used for prior ASCEND expert consensus publications, as it allowed for practical constraints including geographical spread and regional time differences among this international group. The consensus process was conducted in accordance with ACcurate COnsensus Reporting Document (ACCORD) guidelines [20].

The present article discusses and summarizes these recommendations and algorithms, and is intended as a valuable tool to assist countries and individual clinicians in formulating strategies to optimize MCI patient care.

## 3 Recommendations for MCI Assessment and Diagnosis

### 3.1 Challenges and Unmet Needs

A key strategy in tackling the increasing prevalence of MCI is to improve early recognition and diagnosis. Across our collective experience in APAC, and consistent with another report from the region [6], the ASCEND survey results indicated a general lack of confidence and expertise in MCI, particularly in primary care, leading to underdiagnosis, and in some cases, misdiagnosis. Suspicion of MCI is often low, and some providers report difficulty differentiating MCI from dementia or from normal aging, forgetfulness, or depression. Diagnostic challenges include a lack of universally accepted and validated screening/detection tools and biomarkers; a lack of training on administering and evaluating cognitive assessments; time constraints reducing providers' capacity to conduct in‐depth evaluations; and lack of provider reimbursement for cognitive assessments in some countries. Patient‐centered barriers include reluctance to report cognitive issues due to fear, stigma, or denial; and sometimes challenges in accessing medical consultations due to physician availability, geographical challenges, or financial constraints [21].

### 3.2 Strategies to Improve MCI Detection

To address these challenges, the ASCEND Expert Group collectively suggests various strategies to equip and empower providers—particularly in primary care—to improve patient assessment and MCI identification.

First, we suggest culturally appropriate MCI awareness/education programs, including digitally‐driven initiatives targeting both primary care clinicians and the community, to encourage wider screening—particularly when patients have risk factors such as cardiometabolic disease or a history of stroke. Notably in Asia, approximately 80% of patients diagnosed with MCI also present with risk factors for cardiovascular disease, including smoking, obesity, hypertension, dyslipidemia, diabetes, or a history of stroke [6].

**FIGURE 1:**
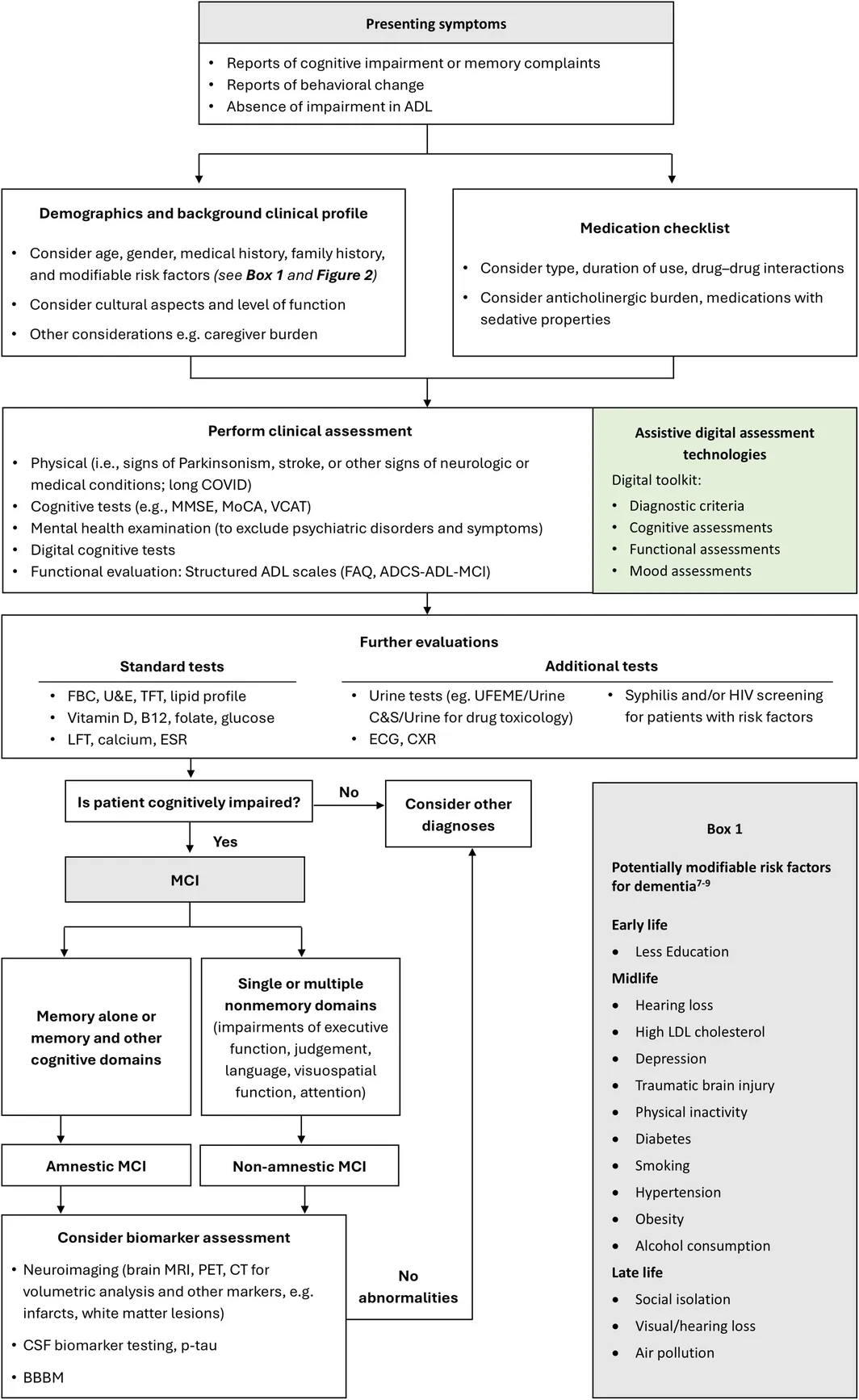
Diagnostic algorithm for mild cognitive impairment. ADCS‐ADL‐MCI, Alzheimer's Disease Cooperative Study Activities of Daily Living Scale for Mild Cognitive Impairment; ADL, activities of daily living; BBBM, blood‐based biomarkers; C&S, culture & sensitivity; CSF, cerebrospinal fluid; CXR, chest x‐ray; ECG, electrocardiogram; ESR, erythrocyte sedimentation rate; FAQ, Functional Activities Questionnaire; FBC, full blood count; HIV, human immunodeficiency virus; LFT, liver function test; MCI, mild cognitive impairment; MMSE, Mini‐Mental Status Exam; MoCA, Montreal Cognitive Assessment; MRI, magnetic resonance imaging; PET, positron emission tomography; p‐tau, phosphorylated tau; STMS, Short Test of Mental Status; TFT, thyroid function test; U&E, urea and electrolytes; UFEME, Urine Full Examination and Microscopic Examination; VCAT, Visual Cognitive Assessment Test.

We recommend increasing resources and capacity to undertake in‐depth consultations at the primary care level. Furthermore, providers need training in standardized, validated diagnostic methods/tools for cognitive screening, memory assessment, MCI diagnosis, referral, treatment/management, and follow‐up. Examples of such tools are presented below and in Figure 1; these may be implemented appropriately according to the country, culture, and institution. Suggested forums for medical education include continuing medical education initiatives, webinars/seminars, workshops, mentorship programs, and multidisciplinary case discussions.

To encourage patients themselves to participate in early screening and assessment, we recommend proactively offering patient self‐evaluation tools including digital cognitive evaluations and AI‐based diagnostic tools.

### 3.3 Assessment and Diagnosis of MCI

Given the QoL challenges associated with MCI and the risk of progression to dementia, we collectively reiterate the importance of timely assessment and diagnosis of MCI to ensure early intervention.

MCI can generally be identified in the primary care setting. However, distinguishing between normal cognitive aging, subjective cognitive impairment (SCI), MCI, and dementia can be a clinical challenge because these states are not strongly delineated, and there is not yet a definitive test for MCI. MCI diagnosis thus requires astute clinical judgment [3]. By consensus, the ASCEND Expert Group has produced a unique diagnostic algorithm for MCI (Figure 1) to guide clinicians in distinguishing and classifying MCI in patients presenting with subjective cognitive or memory complaints.

Initial diagnostic workup should include thorough history‐taking. Relevant medical history includes modifiable risk factors, including comorbidities that are known risk factors for cerebrovascular disease—for example, obesity, hypertension, hyperlipidemia, diabetes, or a history of symptomatic stroke [6, 7]. Additional predisposing factors include hearing loss, vision loss, depression, tobacco use, excessive alcohol consumption, physical inactivity, social isolation, and loneliness (Figure 1—Box 1) [7, 22]. Second, a thorough medication history is important to determine any impact of additive anticholinergic burden, drugs with sedative properties, or other medications known or suspected to affect cognitive function [23]. Third, clinical assessment includes investigation of physical signs of stroke, Parkinsonism, or other neurologic or medical conditions, and mental health examination to exclude psychiatric disorders.

Fourth, cognitive and functional assessments are imperative to distinguish MCI from normal cognitive aging, SCI, or dementia. The MCI diagnosis is based on subjective complaints and/or objective evidence of cognitive decline and memory impairment for age [3], and closely corresponds to the DSM‐5 diagnosis of “Mild Neurocognitive Disorder” [1, 24]. MCI diagnosis generally requires objective assessment of impairment in cognitive domains including executive function, memory, attention, language, and visuospatial skills, but generally without significant impairment in ADL [3]. Validated cognitive assessments include the Mini‐Mental State Examination (MMSE), the Montreal Cognitive Assessment (MoCA), and the Visual Cognitive Assessment Test (VCAT), which was validated in multilingual Asian populations [25, 26, 27, 28]. Structured ADL scales include the Functional Activities Questionnaire (FAQ) and the Alzheimer's Disease Cooperative Study Activities of Daily Living Scale for Mild Cognitive Impairment (ADCS‐ADL‐MCI) [29, 30]. MCI can be categorized as either single‐domain or multiple‐domain, and can be further phenotyped as amnestic or non‐amnestic, based on whether memory impairment is a predominant feature [5].

Finally, laboratory investigations can be performed at the primary care level, including a full blood count, comprehensive metabolic panel, thyroid and liver function tests, urinalysis, lipids, vitamin B12 levels, and tests for relevant infections (Figure 1).

After initial investigations by the primary care physician, patients should be referred for further corroboration and investigations at the specialist level [15]. Blood and cerebrospinal fluid (CSF) biomarker assays are continuing to evolve, albeit encumbered by accuracy, access, and cost concerns. CSF amyloid and p‐tau measurements can help differentiate MCI as part of prodromal Alzheimer's disease (AD) from other forms of dementia [31]. Neuroimaging may also be performed; volumetric markers (e.g., hippocampal volume and medial temporal lobe cortical thickness) and indicators of cerebrovascular disease (e.g., infarcts or white matter lesions) may be useful markers of neuropsychiatric pathology, and are suspected to be associated with progression of MCI to dementia [32, 33, 34]. While the use of biomarker assessments currently lacks definitive consensus [35], results can nevertheless provide useful information regarding pathology, help rule out other causes of cognitive complaints, and inform therapy. We strongly encourage ongoing biomarker research.

### 3.4 Role of Digital Tools in MCI Assessment

In addition to the abovementioned clinic‐based cognitive assessments, most of which can be performed digitally, patient‐executed digital screening tools such as AI‐powered cognitive screening instruments or digitalized assessments can potentially help fill gaps and expedite a diagnosis. The ASCEND Expert Group asserts that digital tools have strong potential utility in clinical practice and advocates for further development and adaptation to maximize their potential. A number of digital diagnostic tools have already been developed, with further examples emerging; for example, the ReCOGnAIze app is a validated, scalable, gamified digital app that effectively detects MCI and vascular cognitive impairment [36].

To help fill additional unmet needs in the diagnosis (and also management) of MCI in Southeast Asia, a novel app‐based toolkit has been developed in consultation with the ASCEND Expert Group, with agreement among the experts regarding aspects to be included. The app is currently undergoing real‐world feasibility testing in six countries (Singapore, Malaysia, Indonesia, Thailand, the Philippines, and Vietnam) as part of the Digital‐based Evaluation and Management in Southeast Asian patients with mild Neurocognitive Disorder (DEMAND) study, with a target enrollment of 1200 patients. This app aims to provide ready access to validated cognitive and functional assessments and diagnostic criteria for MCI, supporting clinicians in their screening efforts, and features a range of post‐diagnosis multi‐domain interventions, discussed in Section 4.4.1, below.

Digital platforms may increase patient engagement, help facilitate early screening by providing standardized step‐by‐step instructions and guidance, and fill existing gaps in screening and diagnosis due to lack of provider resources and time [6]. One systematic review evaluated > 20 digital cognitive assessment tools for MCI and preclinical dementia in older adults. Overall, technology‐based assessments were shown to enhance detection and help facilitate earlier interventions that could potentially lead to improved patient outcomes [37]. Ideally, digital tools may also offer telemedicine options, personalized assistance, and access to disease information.

## 4 Recommendations for MCI Management

Given that MCI is a known risk factor for, and an early manifestation of, dementia [3, 5], early, accurate diagnosis of MCI represents an important opportunity for intervention.

### 4.1 Challenges in MCI Management in APAC

In the pre‐meeting survey, ASCEND experts collectively reported a lack of sufficient confidence among APAC clinicians in managing MCI, particularly in primary care. Key challenges include a lack of training on MCI management and few effective treatment options. In addition, many clinicians experience difficulties in maintaining follow‐up for optimal care, often due to logistical challenges or patient loss to follow‐up.

**FIGURE 2:**
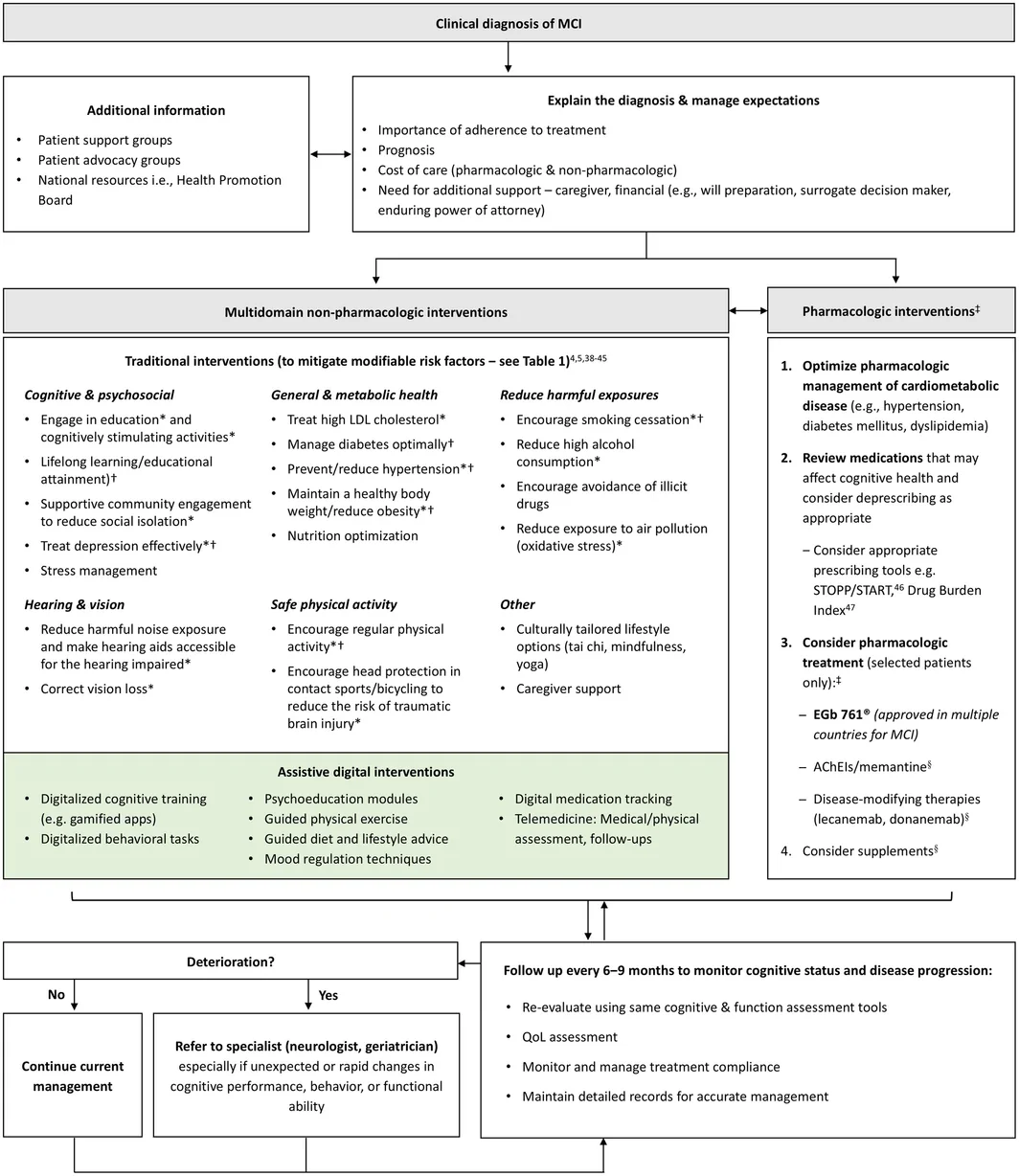
Management algorithm for mild cognitive impairment. *Lancet Standing Commission analysis estimates that almost half of dementia cases may be prevented or delayed by these 14 risk factor modifications [7]. ^†^The World‐Wide FINGERS study (a meta‐analysis of population‐based observational studies from the United States and Europe) concluded that ~30% of AD cases may be attributable to these 7 potentially modifiable risk factors for AD [48]. ^‡^Please refer to local guidelines and prescribe according to approved indications. ^§^Generally not recommended in most guidelines due to lack of evidence in MCI [4, 5, 40, 41, 43, 45]. AChEI, acetylcholinesterase inhibitor, LDL, low‐density liopoprotein; MCI, mild cognitive impairment; QoL, quality of life; STOPP/START, Screening Tool of Older Persons' Prescriptions–Screening Tool to Alert to Right Treatment.

To help increase confidence among providers, and to assist in formulating individualized management plans for patients with MCI, the ASCEND Expert Group has collectively produced a well‐defined management algorithm, presented in Figure 2 and discussed below.

### 4.2 Explanation and Education

After a new diagnosis of MCI, patients and their caregivers need empathy and simple, clear information. It is important to reassure, explain the diagnosis including changes that might be expected over time, and clarify that while MCI can progress to dementia in some individuals, there are interventions that may help prevent or slow further decline as part of a personalized care plan. Primary goals of MCI management are to maintain or improve cognitive function, memory, NPS, and psychological well‐being; to stabilize or delay progression; to preserve independence and QoL; and to alleviate caregiver burden [1]. Early intervention should be encouraged.

### 4.3 Non‐Pharmacologic Approaches—The Mainstay of MCI Management

As mixed pathologies are common in MCI, a holistic, multi‐domain approach with interventions simultaneously targeting multiple modifiable risk factors and mechanisms may be required to achieve optimal benefit [38, 48]. Global studies and guidelines, including from the World Health Organization (WHO), recommend a range of interventions for optimal brain health across the life course (Figure 2, Table 1) [5, 7, 38, 39, 40, 42, 45, 48]. The ASCEND Expert Group asserts that these interventions collectively play a vital role in the prevention and management of MCI.

**TABLE 1:** Guideline comparison of treatment recommendations for MCI symptoms or reducing risk of cognitive decline.

Guideline/Consensus	Non‐pharmacologic management	Drugs e.g., AChEI, memantine	EGb 761	Other supplements
APAC (ASCEND 3 Consensus—present article—refer to Table 2)	Recommended to maintain or improve cognitive function and/or slow cognitive decline: Optimizing general and metabolic health—including through regular physical activity, optimal nutrition, weight control, and vascular risk monitoring Cognitively stimulating activities Treating depression and neuropsychiatric symptoms as appropriate Cessation of smoking and harmful alcohol consumption Remedying hearing and vision loss Encouraging strong social connections and support networks to reduce isolation Reducing exposure to air pollution Optimal management of chronic and cardiovascular diseases Medication review; deprescribing of medications that could exacerbate cognitive symptoms, if possible Use of symptomatic treatments that are efficacious in MCI and well tolerated in older persons	AChEI/memantine: Lack of robust evidence (CoR, III, LoE, A)	Clinically appropriate option for the symptomatic treatment of MCI, as part of a multidomain intervention strategy (CoR, IIA; LoE, A)	Lack of robust evidence in MCI
APAC ASCEND 2 Consensus, 2021 [4]		AChEI: Lack of robust evidence (CoR, III; LoE, A)	Clinically appropriate to use as part of multidomain intervention (CoR IIB; LoE, A) May improve cognitive performance (CoR, I; LoE, A) May help delay dementia in some individuals (CoR, IIB; LoE, C) Favorable risk–benefit profile (LoE, A)	Not mentioned
WHO Recommendations, 2022 [38]	Recommendations to reduce risk of cognitive decline and dementia Cognitive training (QoL, Very low–Low; SoR, Conditional) Physical activity (QoE, Low; SoR, Strong) Nutritional interventions: Mediterranean diet (QoE, Moderate; SoR, Conditional); WHO healthy diet (QoE, Low‐High; SoR, Strong) Tobacco cessation (QoE, Low; SoR, Strong) Cessation of hazardous/harmful alcohol intake: (QoE, Moderate; SoR, Conditional) Midlife weight management (QoE Low–Moderate; SoR, Conditional) Hypertension management (QoE, Low–High; SoR, Strong) Diabetes management (QoE, Very low: SoR, Conditional) Dyslipidemia management (QoE, Low; SoR, Conditinonal) Notes: Interventions for social isolation, depression, and hearing loss were not listed due to lack of data.	Not mentioned	Not mentioned	Not mentioned
AAN Practice Guideline Update, 2018 [5]	Cognitive interventions: May improve select measures of cognitive function (Confidence, Low) Physical activity: 6 months of twice‐weekly exercise training is likely to improve cognitive measure (Confidence, Moderate) Medication management: Consider deprescribing medications that can contribute to cognitive impairment, where feasible and medically appropriate	Donepezil: Possibly ineffective for reducing progression to AD (Confidence, Low) Galantamine: Probably ineffective for reducing progression to AD (Confidence, Moderate) Rivastigmine: Possibly ineffective for reducing progression to AD (Confidence, Low) Piridebil; V0191: Insufficient data (Confidence, Very low) Tesamorelin injections: Possibly effective on cognition (Confidence, Low) Investigational drugs: Clinicians may inform patients about clinical trials (LoE, C)	Not mentioned	Vitamin E: Possibly ineffective for reducing progression to AD (Confidence, Low) Flavonoids; homocysteine‐lowering B‐vitamins; nicotine patches: Not recommended (Confidence, Low or Very low)
AUSTRALIA Recommendations, 2022 [39]	Cognitive activity: Advise to undertake some cognitive activity/remediation and memory training Physical activity: Tailored exercise Address cardiovascular disease: Intervene as early as midlife (obesity, hypertension, hypercholesterolemia, heart disease, diabetes) Optimize nutrition: Mediterranean diet or similar Smoking cessation: Should be recommended Depression: Evaluate; recommend mindfulness practice Impaired hearing: Detect and address Cessation of heavy harmful alcohol consumption Screen for sleep disorders and treat Encourage social engagement Deprescribe medications that impair cognition where possible: Includes those that contribute to cholinergic burden Reduce exposure to air pollution	Investigational drugs: Inform patients of possibility of research participation	Not mentioned	Fortasyn Connect: Patients should be informed of availability
JAPAN Guidelines, 2017 [40]	Moderate physical activity, aerobic exercise: Small studies, insufficient evidence Mediterranean diet, cocoa: Small studies, insufficient evidence Smoking cessation: Small studies, insufficient evidence Cognitive rehabilitation/training: Further studies are needed, but memory support tools are useful Control hypertension, diabetes, dyslipidemia, and cerebrovascular disorders: Appropriate control is recommended, although evidence is insufficient	AChEI and memantine: No recommendations in MCI	Effect of *Ginkgo biloba* has not been confirmed	Effect of vitamin E not confirmed
CHINA Chinese Medical Association Guideline, 2015 [49]			EGb 761 recommended as combination therapy with synthetic drugs, e.g., AChEI (high quality evidence in MCI)	
VIETNAM Alzheimer Disease & Neurocognitive Disorders Association Guideline, 2023 [41]	Exercise: Recommended Cognitive training: May improve MCI cognitive function Control vascular risk factors: Recommended, including smoking cessation Nutrition: Recommend Mediterranean diet, avoid heavy alcohol consumption Correct hearing loss, treat depression, increase social interaction	AChEI: not recommended for MCI	EGb 761 improves cognitive function/memory, NPS and improves QoL in patients with MCI; can be used for MCI with evidence of cerebrovascular disease; may help delay progression	Vitamins/antioxidants: inconsistent results
PHILIPPINES Dementia Society of the Philippines [42]	Endorses adherence to WHO Guidelines [47] and Lancet Commission Report [7] recommendations for multidomain interventions (physical activity, nutrition, tobacco cessation, alcohol reduction, cognitive interventions, social activity, weight management, management of cardiovascular risk factors, depression, hearing/vision loss) Dance intervention may improve cognition, attention, and visuospatial skills		EGb 761 approved for MCI in the Philippines; 120 mg twice daily recommended	Virgin coconut oil (more evidence needed)
SPAIN Spanish Society of Geriatrics and Gerontology Consensus [43]	Cognitive training, stimulation and emotional processing (including digital tools) Support and psychotherapy Music/art therapy; sensory interventions Physical exercise	AChEI and memantine: No evidence to support	EGb 761 may improve behavior, anxiety, executive functions; is the only drug approved for this indication in Spain	Omega‐3 fatty acids may have neuroprotective properties against cerebral atrophy Fortasyn Connect: AD only Citicoline
SWITZERLAND Consensus [44]	Cognitive stimulation, behavioral training Regular physical activity, nutritional advice Social activities Optimal monitoring of cardiovascular risk Music intervention, cooking activities: May lead to positive mood changes		“State of the art” is *Ginkgo biloba* extract 2 × 120 mg/day	
ITALY Guideline, 2024 [45]	Physical exercise: Consider to treat cognitive symptoms and promote independence (Evidence: Weak in Favor) Cognitive training: Consider cognitive training (Evidence, Strong in Favor), games and rehabilitation strategies (Evidence, Weak in Favor) Nutrition: Do not offer ketogenic dietary interventions (Evidence: Strong Against) Creativity: Consider art therapy, dance, music therapy (Evidence: Weak in Favor) Other: Do not offer acupuncture (Evidence, Weak Against) or aromatherapy (Evidence, Strong Against) Do not offer transcranial stimulation (Evidence, Weak Against)	AChEI and memantine: Not recommended due to lack of evidence and AEs mAbs: Under investigation	Do not offer *Ginkgo biloba* (Evidence, Strong Against)	Do not offer supplements, ginseng, antioxidants, or vitamins in the absence of documented deficiencies (Evidence, Strong Against)

Abbreviations: AAN, American Academy of Neurology; AChEI, acetylcholinesterase inhibitor; AChEIs include donepezil, rivastigmine, and galantamine; AD, Alzheimer's dementia; AE, adverse event; CoR, class of recommendation; LoE, level of evidence; NMDA, N‐methyl‐D‐aspartate; QoE, quality of evidence; SoR, Strength of recommendation; WFSBP, World Federation of Societies of Biological Psychiatry; WHO, World Health Organization.

#### 4.3.1 Multi‐Domain Interventions

Firstly, there is evidence to suggest that optimizing general and metabolic health—including through regular physical activity, optimal nutrition, and effective management of cardiometabolic risk factors—may help reduce cognitive decline [5, 38, 39, 40, 42, 45], possibly through benefiting neurodegenerative and cerebrovascular pathologies [4]. While data specifically on physical activity for preserving cognitive function are somewhat inconclusive [51], regular physical activity is recommended by the WHO and other professional societies, including ASCEND, based on overall health benefits [5, 38, 39, 40, 42, 45, 51].

Evidence for cognitive training and rehabilitation is also mixed. ASCEND experts concur with the WHO and other esteemed bodies that cognitively stimulating activities may improve some measures of cognitive function and are worth undertaking with the goal of maximizing cognitive health [5, 7, 8, 38, 39, 40, 42, 45, 49].

Additionally, while there are few studies specifically supporting the benefits of optimizing psychosocial health to prevent cognitive decline [38], the ASCEND Expert Group collectively suggests treating depression appropriately, and encouraging strong social connections and support networks to reduce isolation [7, 39]. Access to counseling services and support groups can also enhance a patient's ability to cope with the emotional and practical aspects of living with MCI.

As recommended in some guidelines, ASCEND advises avoidance of tobacco [7, 38, 39, 40, 42], excessive alcohol [7, 9, 38, 39], and air pollution exposure where possible [7, 39]. Furthermore, we concur with guidelines that recommend correction of hearing and vision loss if required [7, 39, 42].

Culturally appropriate holistic practices are important to many patients across APAC. Although strong evidence is lacking, creative practices such as mindfulness, tai chi, yoga, dance, or music therapy may enhance overall well‐being and could contribute to cognitive health [46, 48, 49, 50]. Patients can try these practices as desired as part of their personalized care plan.

Lastly, we emphasize the importance of caregiver support and education to help alleviate the burden on those who care for loved ones with MCI. The anxiety of impending responsibility and the ongoing burden on caregivers can be substantial, especially when many are themselves of advanced age [52].

#### 4.3.2 Global Evidence for Multi‐Domain Interventions

While two randomized trials of multidomain non‐pharmacologic interventions (Multidomain Alzheimer Preventive Trial [MAPT] [53] and Prevention of Dementia by Intensive Vascular Care [Pre‐DIVA] [54]) did not demonstrate a reduction in incident dementia, the overall evidence for multi‐domain interventions is growing.

The FINnish GERiatric Intervention Study to Prevent Cognitive Impairment and Disability (FINGER) was the first large, long‐term (2‐year) randomized controlled trial to demonstrate that a multidomain lifestyle intervention—including diet, exercise, cognitive training, and vascular risk monitoring—compared with general health advice alone can help improve or maintain cognitive function in older individuals at increased risk of dementia. With these interventions, patients demonstrated improved performance in all cognitive sub‐domains, including executive function, processing speed, and complex memory tasks [8], and these benefits were observed regardless of sociodemographic or socioeconomic factors or other baseline characteristics [55]. The World‐Wide FINGERS Network (WW‐FINGERS) continues to build on these findings, aiming to adapt these interventions across countries and settings, to promote international initiatives for optimizing the care of older individuals [48]. As part of WW‐FINGERS, the SINgapore GERiatric intervention study to reduce cognitive decline and physical frailty (SINGER) study is currently evaluating the effectiveness of the WW‐FINGERS multidomain interventions, with a target enrollment of 1200 older individuals in Singapore who are at risk for dementia [56].

Consistent with the FINGER study outcomes, a recent *Lancet* Standing Commission report based on a comprehensive meta‐analysis estimated that almost half of dementia cases could be prevented or delayed by interventions targeting 14 major risk factors (Figure 2) and concluded that “the potential for prevention is high” [7]. Caution is prudent, as many clinical questions remain unanswered, including the optimal timing and duration of each intervention. Additionally, there are limitations when extrapolating observational findings to interventional effects in the presence of many unmeasured confounders [57].

### 4.4 Role of Digital Tools in Holistic MCI Management

The ASCEND pre‐meeting survey highlighted the important and evolving role of digital assistive technologies and therapeutics in modern healthcare, including in the management continuum for patients with cognitive impairment. In the current digital era, WHO recommends the use of digital innovations to combat dementia risk [38]. As an Expert Group, ASCEND strongly recommends the use of digital platforms and interactive interfaces to (1) expand the use of telehealth for enhanced interaction between patients and care teams, (2) increase patient engagement, (3) provide targeted cognitive training, (4) encourage lifestyle change and medication adherence, and (5) provide linked access to educational materials and community resources. For clinicians, digital tools can assist with patient assessment, help monitor cognitive function through gamified assessments and virtual consultations, inform personalized management strategies, and facilitate shared decision‐making. For caregivers, digital tools that help MCI patients self‐manage/self‐monitor and maintain their independence may alleviate some caregiver burden.

#### 4.4.1 DEMAND Study: Testing a Novel Digital Therapeutics Platform

As mentioned above, the DEMAND study is currently testing the feasibility of a multi‐domain app‐based tool to help fill unmet needs in MCI diagnosis and management in Southeast Asia. DEMAND is enrolling patients who received a diagnosis of MCI and initiated treatment with the *Ginkgo biloba* extract, EGb 761 (discussed below) within the 3 months before recruitment. Patients' baseline cognitive performance will be compared with their performance after 24 weeks of using the app. The platform comprises weekly tasks including cognitive training games, psychoeducation modules, and guided lifestyle advice and exercise videos, with the goals of optimizing cognitive function, supporting patient self‐management, and reducing clinician and caregiver burden. Other measures will include a before‐versus‐after pictorial cognitive test and QoL metrics. Preliminary results of the DEMAND study are promising.

#### 4.4.2 Accessibility and Equality Considerations

It should be noted that not all patients have access to digital devices or internet connectivity, and not all are technologically literate—particularly older persons in developing countries and rural areas. For these patients, low‐tech solutions including phone‐based screening and monitoring, mobile in‐person healthcare and education programs, and paper‐based resources can help ensure patients do not miss out on important assessments, advice, and access to care. Implementation challenges may also exist with digital tools, including those associated with data privacy, cost, and infrastructure barriers.

### 4.5 Pharmacologic Management of MCI

In tandem with non‐pharmacologic interventions, the ASCEND group suggests a three‐pronged approach to the pharmacologic management of MCI (Figure 2).

#### 4.5.1 Optimal Treatment of Chronic Diseases

As discussed above, the mainstay of clinical care for MCI focuses on mitigating modifiable risk factors that contribute to cognitive decline. To help reduce the risk of further decline and dementia, the WHO and other reputable groups recommend optimizing the pharmacologic management of cardiovascular disease, hypertension, dyslipidemia, and diabetes mellitus [7, 38, 39, 40].

#### 4.5.2 Medication Review

Equally important is the avoidance of medications known to contribute to cognitive impairment. Many older adults with MCI or dementia take numerous daily medications [52, 58]. A patient's overall medication load should be screened for aspects that could potentially exacerbate cognitive symptoms, including high anticholinergic burden or the use of drugs with sedating effects [45, 49]. Judicious prescribing and regular medication reviews may help minimize iatrogenic cognitive decline and polypharmacy‐related risks. Deprescribing tools, including STOPP/START [46] or the Drug Burden Index [47], can assist in this process.

#### 4.5.3 Pharmacologic Interventions

Symptomatic treatment of MCI remains an area of active research, but pharmacologic options are presently limited. Therapeutics should be selected according to their tolerability, including in older individuals, and their demonstrated efficacy specifically in MCI. Efficacy in AD or other dementias does not necessarily translate to efficacy in MCI [60].

A number of drugs, including acetylcholinesterase inhibitors (AChEIs; e.g., donepezil, rivastigmine, and galantamine) and memantine are sometimes used off‐label for patients with mild to moderate AD, but these are associated with challenging side effects and have not consistently shown symptomatic benefit in MCI [15, 60, 61, 62, 63, 64, 65]. Multiple guidelines for MCI do not recommend their use due to insufficient evidence [4, 5, 45, 65]. Some evidence, however, suggests that AChEIs may protect against progression from MCI to dementia [60]. Disease‐modifying therapies such as anti‐amyloid monoclonal antibodies (e.g., lecanemab and donanemab) are sometimes used for patients with signs of early AD, but their access is limited in some areas due to difficulties accessing the required imaging assessments [6].

Additionally, a number of supplements have been trialed for MCI. While not recommended in guidelines due to lack of evidence [5, 40, 45], vitamin B and D and Omega‐3 supplements are sometimes used in Asia [6]. A proprietary beverage containing docosahexaenoic acid, eicosapentaenoic acid, uridine monophosphate, choline, phospholipids, selenium, folic acid, and vitamins B12, B6, C, and E has shown some favorable effects on cognition and memory in early AD, but has yet to be robustly tested in MCI [66]. Flavonoids and nicotine patches are not recommended due to lack of evidence [5]. In one small placebo‐controlled study, a Chinese herbal mixture showed some promise in cognitive improvements and delayed progression to AD in patients with amnestic MCI, with improvements in some measures comparable to the widely used EGb 761 [67]; although further studies are required to establish its efficacy.

EGb 761 is a cognitive enhancer registered as a drug for use in MCI across multiple European and Asian countries, including Germany, Switzerland, Mexico, Vietnam, Thailand, and the Philippines. In clinical trials, EGb 761 has demonstrated an ability to stabilize or slow cognitive decline in patients with AD and vascular dementia [68], and shows similar promise in patients with MCI [4, 19, 69]. EGb 761 is recommended for the symptomatic treatment of MCI in several guidelines and consensus publications [4, 19, 41, 43, 44, 49, 70]. Briefly, EGb 761 has been shown to promote brain circulation, protect against oxidative stress, and provide neuroprotective effects, potentially improving cerebrovascular risk [19, 71]. Data from at least eleven randomized trials and several real‐world studies in patients with diagnosed MCI (mostly amnestic) collectively suggest that EGb 761 may enhance cognitive function and reduce NPS [67, 69, 72, 73, 74, 75, 76]. Additionally, some randomized trials and observational studies indicate that EGb 761 may help reduce progression to dementia [15, 77, 78, 79, 80, 81]; though not all studies demonstrate this benefit [82].

A myriad of emerging agents are under investigation specifically for MCI, including AD medications, antiepileptics, antidepressants, cardiometabolic drugs, PDE4 inhibitors, neuroprotective drugs, amyloid‐ and p‐tau protein‐targeting agents, neurotrophics, glutamate receptor modulators, antiretrovirals, and others. In addition, studies are investigating various probiotics, supplements, antioxidants, nutraceuticals, Chinese herbs, and nootropics. Clearly, MCI remains an active area of research. The ASCEND Expert Group suggests that patients be informed of appropriate opportunities to participate in clinical trials that demonstrate strong rationale specifically in MCI [5, 39].

#### 4.5.4 Multidisciplinary Care and Follow‐Up

The ASCEND pre‐meeting survey highlighted a need for multidisciplinary collaboration and integrated care. We assert that, in the overall management of MCI, fostering strong multidisciplinary collaboration between specialists and primary care providers is essential to ensure high‐quality, patient‐centered care. Integrated care teams comprising primary care clinicians, specialists (psychiatrists, neuropsychologists, geriatricians), nurses, social workers, and other healthcare professionals enable the reciprocal exchange of knowledge and best practices, and support the development of clear, standardized protocols for MCI diagnosis, management, and monitoring.

Establishing efficient referral pathways expedites specialist care for tailored recommendations. Patients may be referred back to primary care for follow‐up every 6–12 months to assess cognitive status and monitor progression (Figure 2). Specialists can provide re‐evaluations as needed—for example, if there are rapid or unexpected changes in behavior, functional ability, or cognition. Consistent evaluation tools should be used across visits and clinician types to ensure changes are properly detected over time. This collaborative, shared‐care model improves care continuity and helps optimize patient outcomes. Furthermore, fostering partnerships across private and public healthcare sectors can strengthen MCI care infrastructure and expand access to specialist services.

## 5 Conclusions and Expert Consensus

To supplement the new algorithms presented herein, our expert consensus recommendations regarding MCI diagnosis and management are summarized in Table 2. These expert‐derived consensus recommendations and algorithms have not yet undergone prospective external validation. While we acknowledge the limitations of this document, including the lack of a structured, validated consensus process and possible regional opinion bias limiting international generalizability, our recommendations are well aligned with large international studies [7, 48] and multiple guidelines and consensus publications [4, 5, 6, 38, 39, 40, 41, 42, 43, 44, 45, 49, 65].

**TABLE 2:** Summary of ASCEND expert consensus statements.

**1. MCI Diagnosis**	
1.1 Given the QoL challenges associated with MCI, and the risk of progression to dementia, the ASCEND Expert Group collectively emphasizes the importance of early diagnosis of MCI to ensure timely intervention.	Class of recommendation IIa Level of evidence C (expert opinion)
1.2 We recommend the use of our diagnostic algorithm (Figure 1) as a standardized approach to MCI screening and diagnosis in the APAC region. In primary care, this includes taking a full medical history, medication history, clinical assessment, laboratory investigations, cognitive assessment, memory assessment, and functional assessment (ADL).	Level of evidence C (expert opinion)
1.3 Clinicians should evaluate patients for MCI using validated tools—with consistent use of the same tools across patient visits, to ensure changes in cognitive function are detected.	Level of evidence C (expert opinion)
1.4 Technology‐based cognitive assessments can enhance MCI detection and help facilitate earlier intervention. We urge the ongoing development, refinement, testing, and validation of digital screening and assessment tools.	Class of recommendation IIa Level of evidence A
1.5 After initial investigations by a primary care physician, patients with suspected or diagnosed MCI should be referred for in‐depth corroboration and investigations at the specialist level.	Class of recommendation I Level of evidence C (expert opinion)
1.6 The ASCEND Expert Group encourages ongoing research into blood‐based biomarker testing, including amyloid and p‐tau pathology.	Level of evidence C (expert opinion)
**2. Overall MCI Management**	
2.1 Regardless of their present cognitive status, adult patients should be encouraged to pursue a healthy, active, and socially active lifestyle, and minimize cardiometabolic risk factors that could potentially place them at increased risk of cognitive decline (refer to Statement 2.4 below).	Class of recommendation I Level of evidence C (expert opinion)
2.2 After MCI diagnosis, early intervention is encouraged. Primary goals of MCI management are to maintain or improve cognitive function and psychological/behavioral symptoms, stabilize or delay progression, preserve independence, improve QoL, and alleviate caregiver burden.	Level of evidence C (expert opinion)
2.3 We recommend the use of our MCI management algorithm (Figure 2) as a standardized approach to MCI management in APAC.	Level of evidence C (expert opinion)
2.4 Non‐pharmacologic approaches are the cornerstone of MCI management. In some patients, a multidomain approach targeting risk factors and disease mechanisms may help maintain or improve cognitive function, and/or slow cognitive decline. We recommend the following: Optimizing general and metabolic health—including through regular physical activity, optimal nutrition, weight control, and vascular risk monitoring Cognitively stimulating activities Treating depression and neuropsychiatric symptoms as appropriate Cessation of smoking and harmful alcohol consumption Remedying hearing and vision loss Encouraging strong social connections and support networks to reduce isolation Reducing exposure to air pollution	Class of recommendation I/IIa Level of evidence B/C
2.5 The ASCEND Expert Group advocates for further investment in robust research to build a stronger evidence base for specific interventions that may reduce the risk of cognitive decline.	Level of evidence C (expert opinion)
2.6 The ASCEND Group advocates for digital interventions that provide psychoeducation, targeted cognitive training, healthy lifestyle accountability, and assistance with medication tracking/adherence, to encourage autonomous self‐management, reduce caregiver burden, and assist clinicians with ongoing monitoring of cognitive decline.	Level of evidence C (expert opinion)
2.7 In tandem with non‐pharmacologic interventions, the ASCEND group suggests a three‐pronged approach to the pharmacologic management of MCI: Optimal treatment of chronic and cardiovascular diseases Medication review and deprescribing of medications that could exacerbate cognitive symptoms, where possible Use of symptomatic treatments that: Have proven efficacy in MCI specifically Show good tolerability, including in older persons	Level of evidence C (expert opinion)
2.8 Patients should be followed up with cognitive reassessments every 6–9 months, using the same validated tool to monitor cognitive status and QoL.	Level of evidence C (expert opinion)
**3. Pharmacologic Interventions for MCI**	
3.1 Clinicians should assess for behavioral and neuropsychiatric symptoms in individuals with MCI, and use both non‐pharmacologic and pharmacologic approaches as appropriate	Class of recommendation I Level of evidence C (expert opinion)
3.2 There is a lack of robust evidence supporting the use of AChEI or memantine specifically in MCI symptoms.	Class of recommendation III Level of recommendation A
3.3 Based on evidence from at least 6 randomized trials to date, EGb 761 is a clinically appropriate option for the symptomatic treatment of MCI, as part of a multidomain intervention strategy.	Class of recommendation IIa Level of evidence A
3.3.1 EGb 761 is recommended across multiple guidelines for the symptomatic treatment of MCI.	Class of recommendation I
3.3.2 Based on evidence from randomized studies, and also several prospective observational studies, EGb 761 may also help delay progression of MCI to dementia in some individuals.	Class of recommendation IIa Level of evidence C
3.3.3 EGb 761 has a favorable risk–benefit profile, regardless of patient age.	Class of recommendation IIa Level of evidence A
**4. General Recommendations: Closing Gaps in MCI Care in APAC**	
4.1 We suggest culturally appropriate MCI awareness/education programs targeting both primary care clinicians and the community, to encourage wider screening—particularly when patients have risk factors such as cardiometabolic disease or a history of stroke.	Level of evidence C (expert opinion)
4.2 Providers should undertake training in MCI detection—including administering standardized, validated diagnostic methods/tools for cognitive screening, memory assessment, and MCI diagnosis—and in providing appropriate interventions to reduce the risk of cognitive decline.	Level of evidence C (expert opinion)
4.3 We recommend increasing resources and capacity to undertake in‐depth consultations at the primary care level.	Level of evidence C (expert opinion)
4.4 In the overall management of MCI, fostering strong collaboration and referral pathways between primary care providers and multidisciplinary specialists is essential to ensure high‐quality, integrated, patient‐centered care.	Level of evidence C (expert opinion)
4.5 We advocate for caregiver support and education to minimize caregiver burden.	Level of evidence C (expert opinion)
**KEY:** Class of recommendation Class I: Evidence and/or general agreement (including in multiple professional guidelines) that a given treatment or procedure is beneficial, useful, effective (is recommended/is indicated) Class IIa: Weight of evidence/opinion is in favor of usefulness/efficacy (is reasonable to consider) Class IIb: Usefulness/efficacy is less well established by evidence/opinion (may be reasonable to consider) Class III: Evidence or general agreement that the given treatment or procedure is not useful/effective (is not recommended) **Level of evidence** A: Data derived from multiple randomized, placebo‐controlled clinical trials; meta‐analyses; or recent, well‐conducted systematic reviews including only high‐quality controlled trials B: Data derived from a single randomized clinical trial or large non‐randomized studies C: Consensus of opinion of experts and/or case reports, small studies, retrospective studies	

Abbreviations: AChEI, acetylcholinesterase inhibitors; ADL, activities of daily living; APAC, Asia Pacific; MCI, mild cognitive impairment; QoL, quality of life.

Despite the lack of a definitive test for MCI, our diagnostic algorithm is intended to bolster physician confidence in evaluating patients who present with cognitive complaints or memory symptoms. Regardless of the diagnosis, all individuals—particularly those with risk factors for cognitive decline—should be encouraged to employ the non‐pharmacologic lifestyle interventions described herein as part of a multi‐domain prevention and intervention strategy for cognitive health and overall wellbeing. Among the possible pharmacologic interventions to supplement this approach, growing evidence and clinical consensus suggest EGb 761 may be feasible for treating MCI symptoms and slowing cognitive decline [4, 19, 41, 43, 44, 49, 70].

To support these interventions, the convergence of sophisticated healthcare technology and growing digital literacy among older adults is creating unprecedented opportunities for proactive, accessible, and cost‐effective cognitive health management. Personalizable digital tools are poised to play a crucial role in improving the care experience for patients and caregivers moving forward. Well‐designed tools should seamlessly integrate into patients' daily lives, help them self‐manage and maintain their independence as they actively oppose cognitive decline, and enable better communication between patients, caregivers, and healthcare providers. Collective efforts between physicians and patients, using all the available evidence and tools at their disposal, may ultimately help reduce the burden on healthcare systems while optimizing patient outcomes and QoL.

## Funding

Schwabe Pharma Asia Pacific Pte Ltd. has a written agreement with the ASCEND expert author group to provide funding for advisory board meetings, including the meeting upon which this consensus article was based, and for editorial assistance to prepare this manuscript (no funding ID was necessary). As mentioned above, the manuscript was developed strictly in accordance with GPP guidelines. Schwabe Pharma Asia Pacific Pte Ltd. had no input into the ASCEND meeting content nor the manuscript content, other than checking for factual accuracy.

## Disclosure

ICMJE Authorship Statement: All listed authors have (1) made substantial contributions to the conception or design of this work; and/or the acquisition, analysis, or interpretation of data for this work; (2) been involved in drafting and/or critically revising this work for important intellectual content; (3) provided final approval of the version to be published; and (4) agreed to be accountable for all aspects of the work in ensuring that questions related to the accuracy or integrity of any part of the work are appropriately investigated and resolved.

## Ethics Statement

This manuscript complies with ethical standards and relevant guidelines, as described below. This article is a narrative review and did not involve primary research on human or animal subjects, so did not require ethical approval for the same. The development, review, and editing processes for this manuscript closely followed the principles of the International Society for Medical Publication Professionals (ISMPP) Code of Ethics, adhered to current Good Publication Practice (GPP) recommendations and the International Committee of Medical Journal Editors (ICMJE) authorship criteria (see below). This review complied with the ACcurate COnsensus Reporting Document (ACCORD) reporting guidelines for consensus methods in health‐related research (https://journals.plos.org/plosmedicine/article/file?id=10.1371/journal.pmed.1004326&type=printable) [20] as well as the Scale for the Assessment of Narrative Review Articles (SANRA) checklist for narrative reviews (https://pmc.ncbi.nlm.nih.gov/articles/PMC6434870/pdf/41073_2019_Article_64.pdf).

## Conflicts of Interest

All authors participated in the Expert Panel from which this document was drafted, and each critically reviewed and approved the manuscript. Other than participating in the Expert Panel, the following authors declare no conflicts of interest: Si Ching Lim, Tong Mai Trang, Yuda Turana, Vorapun Senanarong, and Ralf Ihl. Other authors declare the following, pertaining to the past 3 years: Nagaendran Kandiah is a scientific advisor to Neurophet and SGDiagnostics. He has received honoraria for speaking engagements and advisory board meetings for Eisai, Eli Lilly, Roche, Schwabe and Danone. Chan Yee Fai has received honoraria from Lundbeck and Otsuka; CME sponsorship from Menarini and EP Plus Group; lecture fees from Lundbeck and Otsuka; and was involved as a consultant, speaker, or in advisory boards for Menarini, Lundbeck and Otsuka, Eisai. D Darwin A. Dasig has received honoraria from Lundbeck Philippines Inc. and Menarini Philippines; CME sponsorship from Lundbeck Philippines Inc., Menarini Philippines, E* Chimes Pharmaceutical Inc., and Hi Esai Pharmaceutical Inc.; lecture fees from Lundbeck Philippines Inc. and Menarini Philippines; and was involved as a consultant, speaker, or in advisory board for Lundbeck Phillipines Inc. and Menarini Philippines. Jacqueline Dominguez has received lecture fees from Otsuka Philippines. Kar Chai Koh has received honoraria in Malaysia from Takeda, GlaxoSmithKline, Reckitt Bengkiser, Abbott Diagnostics, Sanofi Aventis, Novo Nordisk, Mundipharma Pharmaceuticals, Xepa Soul Pattinson, EP Plus Group, Novartis, Pfizer, and Zuellig Pharma. Paulus Anam Ong has received honoraria from Moleac Pharma and CME sponsorship from Darya‐Varia Pharma, and has been involved as a consultant and speaker for Lundbeck Pharma. Encarnita Raya‐Ampil has received CME sponsorship from Hi Eisai, Menarini, Lundbeck, Otsuka, Medichem, and Torrent; and lecture fees from Hi Eisai, Menarini, Lundbeck, Otsuka, and Medichem. Raymond Seet Chee Seong receives grant funding from the National Medical Research Council, Singapore. Kit Mun Tan has received CME sponsorship from Pfizer and Menarini; and was involved as a consultant, speaker, or in advisory boards for Pfizer, Menarini, Boehringer Ingelheim, Pharma Nord, Novartis. Novo Nordisk, Astra Zeneca, Bayer, and GlaxoSmithKline. Tan Maw Pin has received honoraria from Menarini, Pfizer, Merck Sharp & Dohme, GlaxoSmithKline, Boerhinger Ingelheim, Novartis, Novodisk Nordisk, Moleac, Sanofi, and Abbott; CME sponsorship from Sanofi and Pfizer; lecture fees from Pfizer, Sanofi, GlaxoSmithKline, Novartis, Moleac, Merck Sharp & Dohme, Boerhinger Ingelheim, Abbott, and Menarini; research funding from Sanofi; and has been involved as a consultant, speaker, or in advisory boards for Moleac, Menarini, Sanofi, Pfizer, MSD, Eisai, and GlaxoSmithKline. Tran Cong Thang has received honoraria from Gigamed, Abbott, Everpharma, Menarini, Eisai, Piere Fabre, and Worwag, and has been involved as a consultant, speaker, or in advisory boards for Eisai. Cuibai Wei has received research funding from Eisai, and research grants from Natural Science Foundation of China, the Ministry of Science and Technology of the People's Republic of China, and Jinan Municipal Health Commission Department of Technology and Science of Shandong Province.

## Data Availability

The data that support the findings of this consensus article are available in the public domain (see reference list). Data sharing is not applicable to this article as no datasets were generated or analyzed.
